# Improving retention in HIV care among adolescents and adults in low- and middle-income countries: A systematic review of the literature

**DOI:** 10.1371/journal.pone.0184879

**Published:** 2017-09-29

**Authors:** Kate R. Murray, Lisa S. Dulli, Kathleen Ridgeway, Leila Dal Santo, Danielle Darrow de Mora, Patrick Olsen, Hannah Silverstein, Donna R. McCarraher

**Affiliations:** 1 Global Health, Population, & Nutrition, FHI 360, Durham, North Carolina, United States of America; 2 Global Health, Population, & Nutrition, FHI 360, Washington, DC, United States of America; 3 Maternal and Child Health, Gillings School of Global Public Health, University of North Carolina at Chapel Hill, Chapel Hill, North Carolina, United States of America; ARGENTINA

## Abstract

**Introduction:**

Adolescents living with HIV are an underserved population, with poor retention in HIV health care services and high mortality, who are in need of targeted effective interventions. We conducted a literature review to identify strategies that could be adapted to meet the needs of adolescents living with HIV.

**Methods:**

We searched PubMed, Web of Science, Popline, USAID’s AIDSFree Resource Library, and the USAID Development Experience Clearinghouse for relevant studies published within a recent five-year period. Studies were included if they described interventions to improve the retention in care of HIV-positive patients who are initiating or already receiving antiretroviral therapy in low- and middle-income countries. To assess the quality of the studies, we used the NIH NHLBI Study Quality Assessment Tools.

**Results and discussion:**

Of 13,429 potentially relevant citations, 23 were eligible for inclusion. Most studies took place in sub-Saharan Africa. Only one study evaluated a retention intervention for youth (15–24 years); it found no difference in loss to follow-up between a youth-friendly clinic and a family-oriented clinic. A study of community-based service delivery which was effective for adults found no effect for youths. We found no relevant studies conducted exclusively with adolescent participants (10–19 years). Most studies were conducted with adults only or with populations that included adults and adolescents but did not report separate results for adolescents. Interventions that involved community-based services showed the most robust evidence for improving retention in care. Several studies found statistically significant associations between decentralization, down-referral of stable patients, task-shifting of services, and differentiated care, and retention in care among adults; however, most evidence comes from retrospective, observational studies and none of these approaches were evaluated among adolescents or youth.

**Conclusions:**

Interventions that target retention in care among adolescents living with HIV are rare in the published literature. We found only two studies conducted with youth and no studies with adolescents. Given the urgent need to increase the retention of adolescents in HIV care, interventions that are effective in increasing adult retention in care should be considered for adaptation and evaluation among adolescents and interventions specifically targeting the needs of adolescents must be developed and tested.

## Introduction

In order to eliminate new HIV infections by the year 2030, UNAIDS has put forth the 90-90-90 treatment targets where, by 2020, 90% of people living with HIV (PLHIV) will know their status, 90% of people with a diagnosed HIV infection will receive sustained antiretroviral treatment (ART) for HIV, and 90% of those on ART will achieve viral load suppression [1]. However, at each step along the HIV treatment cascade (the sequential steps of medical care for PLHIV)—HIV testing, diagnosis, linkage to health care services, retention in care, ART adherence, and viral suppression—substantial proportions of PLHIV are lost to follow-up [2]. Adolescents living with HIV are increasingly being recognized as a vulnerable population in need of targeted programs and resources [3–5].

Adolescents experience greater vulnerability to HIV and their performance along the HIV treatment cascade is worse than other age groups. Thirty per cent of new HIV infections occurred among adolescents and youth (15–24 years) in 2014, and HIV is the second leading cause of death among adolescents globally [6, 7]. In fact, health outcomes have been worsening among the 2.1 million adolescents, ages 10–19, living with HIV, 82% of whom reside in sub-Saharan Africa [8]. While HIV-related mortality declined in other age groups between 2005 and 2012, adolescents living with HIV (ALHIV) experienced a 50% increase in mortality during this time [1]. Adolescents are faring poorly along the HIV treatment cascade, with attrition occurring at each step along the cascade [9, 10]. A large proportion of adolescents are unaware of their HIV status and once diagnosed, ALHIV experience poor linkage to care [9–11]. Retention in HIV care is substantially lower for adolescents than for other age groups [12–16]. Finally, adolescents have suboptimal adherence and lower rates of viral suppression compared to other ages [17–21].

Retention in HIV health care services is a critical precursor to ART adherence and viral suppression. Clinical visits for patients on ART are essential to initiate ART, ensure continuous access to medication, monitor medication side effects, diagnose treatment failure, and, when necessary, switch to second- or third-line ART regimens. Retaining patients in care helps them maintain high medication adherence, thereby achieving viral suppression, improving health outcomes, and reducing the risk of horizontal transmission [22, 23]. PLHIV who are not retained in care stop or interrupt ART, increasing their risk of drug resistance and mortality [24, 25]. Retaining patients in care over time is problematic. A recent systematic review of 154 adult HIV patient cohorts in low- and middle-income countries (LMIC) found that, on average, 83% of patients had been retained in HIV treatment services after 12 months on ART, but the proportion declined to only 60% after 60 months on ART [26]. Comparable data on long-term retention in care are not available for adolescents or youth. Data from four LMIC, however, show that youth (15–24 years) had 59% higher attrition than adults (25–54 years) one year after initiating ART underscoring the need to develop and test interventions to improve retention that specifically target youth [12]. As countries begin to implement the WHO recommended “Test and Treat” strategy [27], all individuals who test HIV positive will immediately initiate ART, substantially increasing the number on ART. The challenges of retaining patients in care will likely only amplify with as the number of PLHIV on ART increase, in the absence of effective interventions.

Given the limited literature on interventions for adolescents in LMIC, we included literature describing interventions that were conducted with adults to form recommendations for interventions that showed promise for adaptation for adolescents. The objectives of this systematic review were to (1) identify recent interventions that increase retention in care among adults and adolescents, (2) describe the body of literature in both populations, and (3) identify intervention strategies that were successful among adults that may be adapted to adolescent populations. Our intention is that recommendations from this review will inform future research and programming among adolescents living with HIV in LMIC.

## Methods

We conducted a systematic review of the peer-reviewed and grey literatures to identify studies describing interventions designed to increase either retention in care or adherence among adult and adolescent PLHIV in LMIC settings. The search strategy was adapted from a systematic review of service delivery interventions for ALHIV [28] (S1 File). We modified the search strategy to include adults in order to evaluate the evidence for interventions among these two populations. We used this strategy to search PubMed, Web of Science, and Popline. We also identified grey literature using USAID’s AIDSFree Resource Library [29] and the USAID Development Experience Clearinghouse (DEC) [30].

All references from PubMed, Web of Science, and Popline were imported into EndNote. Grey literature references from the two websites were exported into an Excel file and added to EndNote during the full text review stage. After duplicate references were removed, two reviewers (KM, KR) independently examined titles and abstracts for inclusion. Next, the full texts of the remaining studies were obtained and each was independently examined by teams of two reviewers (KM, KR, DRM, DD, LD, PO) for inclusion. We also hand-searched reference lists of articles captured by the searches to identify other studies of potential relevance and no additional references were found. At each stage of the review, discrepancies between reviewers were resolved through discussion and consensus. Summaries of each article were elaborated by single reviewers (KM, KR, LCD, PO, HS) using a standardized form that extracted information including study design, setting, sample characteristics, sample size, description of the intervention, and outcome results.

A study was eligible for inclusion if it met all the following criteria: (1) evaluated the effects of or associations between an intervention or program and retention in care or ART adherence among HIV-positive patients; (2) reported quantitative measures of retention in care or ART adherence; (3) conducted among adults (age ≥ 18) or adolescents (mean age 10–19); and (4) published within the five-year search period (20 November 2010 to 20 November 2015). Given the rapidly changing environment in which HIV programs operate, we restricted the search to five years in order to capture interventions conducted in the current landscape of HIV programs. This article focuses on interventions to improve retention in care.

We found considerable heterogeneity in the way retention was measured, thus we considered multiple retention outcomes to meet the inclusion criteria, including: retention in care (alive and on ART at a specified timepoint), loss to follow up (LTFU) (not retained in care at a specified timepoint), attrition from care (not retained in care due to death, LTFU or default), and appointment attendance (number of attended appointments out of total possible).

Studies were excluded if they were not available in English or if they were conducted in high-income countries (as defined by the World Bank) [31]. Letters, editorials, conference abstracts, and presentations were not eligible. We also excluded studies tailored to the needs of specific populations (e.g. key populations, patients co-infected with tuberculosis and HIV, or incarcerated populations), pharmaceutical interventions (such as drug regimen changes), post-exposure prophylaxis, HIV testing, linkage to care, and pre-ART care.

The methodological quality of each study was evaluated by a team of six reviewers (KM, KR, PO, LDS, HS, LD) with the aid of the National Institutes of Health National Heart, Lung, and Blood Institute Study Quality Assessment Tools using standardized forms [32]. Two reviewers independently reviewed the quality of each study. Discrepancies in quality assessments were discussed and resolved, with additional review by DRM and LD as needed. The studies were assigned a final quality rating of “good”, “fair” or “poor” to describe the risk of bias in the study due to flaws in study design or implementation. A “good” rating has the least risk of bias, “fair” rating indicates that the study is susceptible to some bias but not sufficient to invalidate results, and “poor” rating indicates significant risk of bias.

## Results and discussion

**Fig 1:**
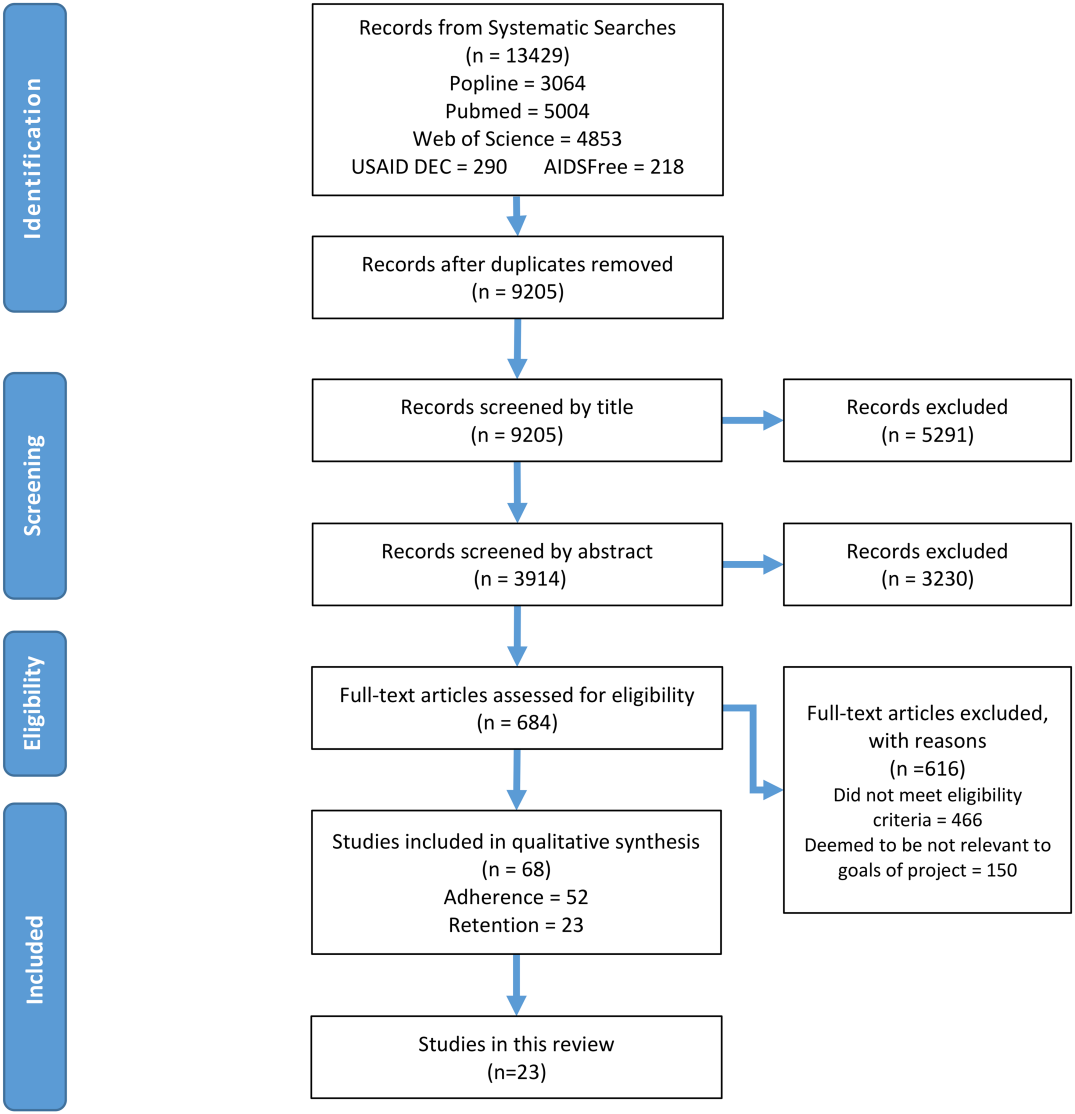
Selection process for inclusion of studies.

A total of 13,429 potentially relevant citations were identified, of which 23 were eligible for inclusion (Fig 1). Most of the included studies were observational. Eleven of the studies were conducted among adults only, 11 were conducted with populations that included adolescents and adults (only one study reported results for youth ages 16–24 years separately), and one study was conducted among youth only (15–24 years). The majority (n = 22) were conducted in sub-Saharan Africa (seven of these in South Africa and six in Kenya) and one study was conducted in Papua New Guinea. We identified 18 retrospective cohort studies, two prospective cohort studies, one quasi-experimental study, and two randomized controlled trials. The methodological quality of the studies varied: nine studies were good quality, nine were fair quality, and five were poor quality.

Among the articles reviewed, most interventions acted at the level of health service delivery. Only two articles described an intervention strategy that acted on the policy level (free or low-cost ART) and one focused exclusively on providing instrumental social support at the individual patient level. The most common intervention types were community-based service delivery (n = 8), decentralization of health services (n = 4), and down-referral (n = 3) (Table 1). Other interventions included task-shifting (n = 2), free or low-cost ART (n = 2), differentiated care (n = 2), patient tracking (n = 1), and instrumental support (n = 1). The only intervention that was conducted exclusively with youth was a youth-friendly differentiated care intervention.

**Table 1:** Summary of intervention types and inclusion age of study populations.

Level	Intervention type	Definition	Youth (15–24) only	Adults + Adolescents	Adults only (18+)	Total
**Policy**	Free/low-cost ART	Policy shift to provide free or low-cost ART to HIV+ patients	0	2	0	**2**
**Health service delivery**	Community-based service delivery	Shifting health care and/or ART distribution to the community (as opposed to health facility); community health workers or peers distribute ART and monitor symptoms; which results in fewer clinic visits	0	4	4	**8**
	Decentralization	Shifting health service delivery from a higher-tier health facility (such as a regional hospital) to a lower-tier facility (such as a primary health clinic)	0	2	2	**4**
	Down-referral	Shifting the delivery of ART for stable patients on ART only from higher-level facilities (such as a hospitals) to lower-level facilities (such as a primary care clinic) to allow hospital staff to focus on ART initiation and managing complicated cases; may also involve task shifting and fewer clinic visits or less frequent ART refills; down-referred patients can be up-referred back to a central facility/provider if they require additional clinical care (due to opportunistic infections or changes in treatment regimen)	0	0	3	**3**
	Task-shifting	Shifting responsibilities for HIV treatment and care from physicians to other health care workers, such as clinical officers and nurses	0	2	0	**2**
	Differentiated care	Service-delivery models that are modified to address the specific requirements of a subgroup of clients	1	1	0	**2**
	Patient tracking	Health clinic staff member (or other designated person) contacts patients after they miss a clinic appointment (either by telephone or home visit) to request that they return to clinic	0	0	1	**1**
**Individual**	Instrumental support	Tangible forms of social support such as nutrition or education support; nutrition-support interventions provide supplementary nutritional to individuals or households; education-support interventions provide financial support for the education of children of PLHIV	0	0	1	**1**

In this review, retention in care is defined and measured in a number of ways in LMIC, presenting a challenge to summarizing the evidence for the interventions in this review. Retention in care can be measured using appointments attended over time, however, in LMIC, retention is often defined as being “alive and on ART” at a specified timepoint per clinical records [33, 34]. Outcomes reported by studies in this review include retention in care, LTFU, attrition from care, and appointment attendance, although the duration of outcome measures varied among the studies. Characteristics of the 23 studies presented in this review are summarized in Table 2 and described in detail below.

**Table 2:** Characteristics of included studies.

Author, year	Country	Study design	Target Population	Sample size	Intervention	Comparison	Outcome measure and definition	Results
Fatti et al., 2012 [37]	South Africa	Retrospective cohort study	HIV-positive adult patients ≥16 years; median age 35.1 years (IQR 29.4–42.3) for exposed; 34.6 years (IQR 29.3–41.4) for non-exposed	Exposed (CBAS) n = 19,668; non-exposed (no CBAS) n = 47,285	Community-based adherence support (CBAS) workers (CHW) conduct weekly home visits for 1 month to perform adherence checks and to provide psychosocial support. Stable patients were visited every 3 months.	Patients who did not receive CBAS	LTFU (no visits to clinic for ≥ 180 days)	Lower LTFU was observed in CBAS patients compared with non-CBAS patients after 5 years in treatment [aHR 0.63, 95% CI (0.59, 0.68)].
Franke et al., 2013 [38]	Rwanda	Prospective cohort study	HIV-positive adults initiating ART; median age: 37 years (range: 21–80)	Exposed n = 304; non-exposed n = 306	Addition of community-based accompaniment (CBA) consisting of daily home visits and DOT by a CHW, nutritional support, transportation stipends, and as-needed socioeconomic support to facility-based care	Facility-based care only	Rate of attrition from treatment [death, LTFU (not returned to clinic for ≥60 days) or default (stopped treatment for ≥60 days)]. Retention (alive and not >60 days since last visit) with viral load suppression.	Receiving CBA was associated with a lower rate of attrition in 1st year of ART [aHR 0.17 (95%CI (0.09, 0.35)].CBA patients were more likely to be retained in care with suppressed viral load at 1 year [aRR 1.15, 95%CI (1.03, 1.27)].
Hickey et al., 2015 [39]	Kenya	Quasi-experimental two-group pre-test post-test study	Patients enrolling in ART; mean age: 39 years in intervention; 40 years in control	Intervention n = 153; control n = 216	Community based microclinics: new ART patients invited to form patient-defined support network, “microclinics”, comprised of close family, friends, or other individuals irrespective of HIV status. Microclinics were assigned a CHW coordinator and participated in 10 biweekly discussion sessions.	Three communities that did not have the micro-clinic intervention.	Clinic absence of ≥ 90 days (days between missed visit and date of return to any clinic) in 22-month period after ART initiation	Intervention participants had one-half the rate of clinic absence ≥ 90-day compared to those in the control arm [HR 0.48, 95% CI (0.25, 0.92)].
Selke et al., 2010 [41]	Kenya	Cluster RCT	HIV-positive, clinically stable patients ≥18 years on ART for ≥3 months; mean age 38.7 years in intervention; 37.5 years in control	Intervention n = 96; control n = 112	Community Care Coordinators (lay PLHIV with secondary school education) conducted monthly home visits with patients and used PDAs to collect data on symptoms, vital signs, and adherence and distributed a monthly supply of ART to patients in their homes. Intervention participants also attended clinic visits every 3 months.	Standard of care includes monthly clinic visits with health care provider, 1-month supply of all medications.	LTFU (not defined)	No difference in LTFU at study closure: 5.2% in intervention group compared to 4.5% in control group (p = 1.0).
Grimsrud et al., 2015 [42]	South Africa	Retrospective cohort study	HIV-positive clinic patients ≥16 years initiating ART from 2002–2012; median age: 33.4 years (IQR 28.4–39.8)	Exposed n = 2,113; non-exposed n = 6,037	Community-based adherence clubs (CACs): CACs consisted of 25–30 stable (self-reported adherence to ART, on ART for >12 months, 2 consecutive suppressed VL (<400 copies/mL), no active opportunistic infections (OIs)) HIV patients who formed a community-based support group led by CHW and supported by nurses. CAC met every 2 months for counselling, symptom screening, and pre-packaged ART distribution and conducted one clinical consultation per year. Patients who develop complications are referred back to the CHC.	Patients receiving ART at community health centre	LTFU (no visit in first 12 weeks of 2014 or censored after last visit if before then)	CAC participation associated with 67% reduction in LTFU compared with CHC [aHR 0.33, 95% CI (0.27, 0.40)]. No significant difference was found in LTFU for youth (16–24 years old) in CAC vs. CHC [aHR 0.68, 95%CI (0.37–1.22)].
Igumbor et al., 2011 [40]	South Africa	Retrospective cohort study	HIV-positive patients (0–50 + years) initiating ART; mean age and range not reported	Exposed (sites with PA services) n = 540; non-exposed (sites without PA services) n = not reported	Community-based adherence support provided by patient advocates (PAs) who provide psychosocial assessments, treatment education, home visits, and follow-up.	Sites that do not have PA services.	Non-retention in care [LTFU (not defined) or death].	Patients at sites with PA services had lower non-retention in care [HR 0.62, 95%CI (0.62, 0.68)].
Bemelmans et al., 2014 [35]	Malawi	Retrospective cohort study	HIV-positive adults > 15 years on 1st line ART >12 months at 10 health centres in study district; mean age not reported	Sample size not reported	Community-based service: Six-monthly appointment (SMA) protocol allows stable (on first line ART ≥12 months w/ CD4 count ≥300, without OI/side effects, pregnancy or breastfeeding) patients to attend clinic every 6 months instead of 1–2 months. CHWs provide ART refills every 3 months and refer patients to clinical staff as necessary.	Patients who are eligible for but not enrolled in SMA system	Retention (total number of patients on ART care followed in the program) at 36 months after program enrolment	94.3% of exposed patients were retained in care 36 months after enrolment compared to 83.0% of patients eligible for but not enrolled in the program.
Estopinal et al., 2012 [36]	Zambia	Retrospective cohort study	ART-enrolled patients > 18 years at time of initiation; median age: 35.3 years (IQR 30.8–43.5) for exposed; 38.2 years (IQR 31.9–45.7) for non-exposed	Exposed (live in village with HBC) n = 84; non-exposed (live in villages without HBC) n = 439	Community-based service: home-based care (HBC) volunteers provide community education, patient referral, adherence counselling, defaulter tracing, and nutritional support.	Standard of care	Alive and on ART	Availability of HBC had no effect: 80% of intervention group was alive and on ART compared with 82% of control group (p = 0.6).
McGuire et al., 2012 [46]	Malawi	Retrospective cohort study	HIV-positive adult and adolescent patients initiating ART (Included all patients at clinics and 80% of patients were >25 years)	Exposed n = 11,090; non-exposed n = 4,322	Decentralized HIV care was provided by mobile teams in 10 peripheral health facilities starting in 2003. In 2007, nurses at the peripheral health facilities began initiating patients on ART and providing clinical monitoring.	Hospital-based care	Attrition [deaths and LTFU (missed appointment by >2 months)]	2-year attrition lower in decentralized care compared to hospital-based care [9.9 per 100 person-years, 95% CI (9.5, 10.4) vs. 20.8 per 100 person-years, 95% CI (19.7, 22.0)].
Das et al., 2014 [43]	Papua New Guinea	Retrospective cohort study	HIV-positive adult patients initiating ART; mean age: 32 years for exposed; 35 years for non-exposed	Exposed n = 993; non-exposed n = 1,464	Care was decentralized through PAPUA (Patient and Provider Unified Approach) model from regional hospitals to rural health district facilities and coordinated patient and provider support, including case management and material support to patients and clinical mentorship to providers.	Standard of care (not decentralized)	Attrition from care at 12, 24, 36, and 48 months (visit within 90 days of chart review)	PAPUA model associated with 15% lower rate of attrition during the first 4 years of ART compared to standard of care [HR 0.85, 95% CI (0.74, 0.99)].
Labhardt et al., 2013 [45]	Lesotho	Retrospective cohort study	HIV-positive patients ≥16 years initiating ART; median age: 38 years (IQR: 31–48)	Exposed n = 2,042; non-exposed n = 1,705	Care was decentralized from hospitals to health centres, ART provision by nurses was scaled up, and lay counsellors were employed to provide HIV counselling and testing, adherence monitoring, and track patients who were LTFU.	Hospital care	Three-year retention in care (alive on ART and in active follow-up at database closure)	Overall 3-year retention in care was 68.8% (95% CI: 65.7, 71.6) in HCs and 64.1% (95% CI: 61.1, 66.9). 3-year retention in care was similar in HCs and hospitals among women [OR 0.89, 95% CI: 0.73, 1.09) and higher retention at HCs among men (OR 1.53, 95% CI (1.20, 1.96)].
Gorman et al., 2015 [44]	Kenya	Retrospective cohort study	HIV-positive patients living in West Pokot accessing care in mobile clinics or regional hospital; mean age: 36.0 years for exposed; 33.5 years for non-exposed	Exposed n = 124; non-exposed n = 54	Decentralized care: semi-mobile clinic model employed clinical team of ≥ 1 nurse, 1 clinical officer, 1 social worker who delivered care weekly at each health clinic, which were located closer to patients’ homes than the district hospital. HIV-positive patients registered at hospital and were offered choice of continuing care at hospital or at a semi-mobile clinic.	Hospital-based care	Retention in HIV treatment (ratio of # scheduled monthly visits attended to total # months in treatment)	Retention did not differ significantly between the two groups. Mean proportion of visits attended was 77% for semi-mobile clinic and 71% for hospital clinic (p = 0.2).
Luque-Fernandez et al., 2013 [50]	South Africa	Retrospective cohort study	HIV-positive adult patients ≥18 years; median age: 33 years (IQR: 29–39)	Exposed n = 502; non-exposed n = 2,327	Down referral of clinically stable (on ART for at least 18 months, CD4 >200 cells/μL in previous 6 months, have sustained viral load suppression) patients to a group-based model of care (adherence club). Groups of 15–30 patients met at the health clinic outside of busy clinic hours, trained counsellors led group discussions, conducted health assessments and provided clinic referral as needed. Patients received individually packaged medicines, and VL and CD4 tested annually by a nurse.	Patients who received routine nurse-led care in the health clinic	Attrition [Death or LTFU (not having any contact with service in 6 months following analysis closure)]	Adherence club participants had lower odds of attrition [HR 0.43, 95% CI (0.21, 0.91)] between 2007–2011.
Brennan et al., 2011 [48]	South Africa	Retrospective matched cohort study	Stable HIV-positive patients ≥18 years; median age: 35.3 years (IQR: 30.8–41.6)	Exposed n = 693; non-exposed n = 2,079	Down-referral of stable (on ART for at least 11 months, no OIs, CD4>200 cells/μL, stable weight (<5% loss between last three visits), virally suppressed (two consecutive viral loads <400 copies/ml)) patients from doctor-managed treatment initiation site to nurse-managed primary health clinic. Patients who were down-referred also received 2 month supplies of ART and clinic visits were held every 2 months.	Patients who remained at the doctor-managed treatment initiation site. Eligible patients who were not down-referred had either refused or never been offered down-referral, but data does not distinguish between these two.	LTFU (≥ 3 months late for last scheduled visit)	LTFU was lower for down-referred patients than those who remained at treatment initiation site [HR 0.3, 95% CI (0.2, 0.6)] during 12 month follow up.
Grimsrud et al., 2014 [49]	South Africa	Retrospective cohort study	HIV-positive adult patients eligible for ART (based on national guidelines); median age: 34 years (IQR: 29–40)	Exposed n = 2,341; non-exposed n = 2,234	Down referral of stable (on ART for at least 16 weeks, most recent viral load <50 copies/ml, no active OIs or poorly controlled chronic conditions, on a first-line ART regimen, and demonstrated good adherence by pill count) patients to nurse-managed clinical care every 4 months at separate building on grounds of same clinic as the doctor-managed clinic. Patients were dispensed 2-month supply of ART through pharmacy.	Patients at the doctor-managed clinic	LTFU (no contact in 6-month period between end of analysis and database closure)	Down-referred patients had slightly higher risk of LTFU compared to those at doctor-managed clinic [aHR 1.36, 95% CI (1.09, 1.69)].
Fairall et al., 2012 [51]	South Africa	Cluster RCT	HIV-positive patients ≥ 16 years; median age 36 years (IQR 30–43) for intervention; 38 years (IQR 29–42) for control)	Intervention n = 5,390; control n = 3,862	Task shifting through Streamlining Tasks and Roles to Expand Treatment and Care for HIV (STRETCH): education and training for nurses to initiate and re-prescribe ART in order to shift the responsibility for ART initiation and management from doctors to primary care nurses.	Standard care	Program retention (alive and in care, with documentation of clinic visit or lab test in previous 6 months) at 12 months after enrolment into the study	Program retention higher for patients newly initiating ART in the intervention group than in the control group (RR 1.10, 95% CI (1.04, 1.16)). No difference in program retention was seen for patients who had been on ART for at least six months when the intervention was introduced.
McGuire et al., 2013 [52]	Malawi	Retrospective cohort study	HIV-positive adults > 15 years who had more than 1 follow-up visit after ART initiation; median age: 35.1 years (IQR 29.4–43.1)	Clinical officer care n = 13,386; nurse care n = 1,901; mixed nurse and clinical officer care n = 4,825	Task shifting: patients received care either from clinical officers (>80% of care), nurses (>80% of care), or both (<80% of care from either nurses or clinical officers). Clinical officers managed both complicated and uncomplicated patients. Nurses primarily managed less complicated patients. Mixed group (nurses and clinical officers) managed patients according to their evolving clinical status.	Clinical officer group	Program attrition [LTFU (time period not defined) and mortality] at 2 years after ART initiation	Compared to nurse care group, attrition was higher in clinical officer group [aIRR 3.03, 95% CI (2.56, 3.59)] and lower in mixed care group [aIRR 0.54, 95% CI (0.45, 0.65)]
Braitstein et al., 2012 [53]	Kenya	Retrospective cohort study	HIV-positive patients ≥ 14 years initiating ART with CD4 counts of < 100 cells/mm^3^; mean age: 36 years (IQR 30.5–42.4) for exposed; 37 years (IQR 30.6–43.1) for non-exposed	Exposed n = 635; non-exposed n = 4,323	Differentiated care through High Risk Express Care (HREC) intervention consisting of weekly or bi-weekly rapid contacts with nurses in addition to routine care for patients initiating ART with low CD4 counts during first 3 months of ART initiation. HREC nurses ask about adherence, conduct pill count, review symptoms, and performs interim clinical assessments, referring patients to clinical officer/physician if indicated.	Routine care consisting of a clinician visit (clinical officer or physician) 2 weeks after initiating treatment, and monthly thereafter.	LTFU (patient did not return to clinic for > 3 consecutive months following most recent visit)	Patients in HREC had lower LTFU [aHR 0.62, 95% CI (0.55, 0.70)] compared to patients in routine care.
Ojwang et al., 2015 [54]	Kenya	Retrospective cohort study	HIV-positive youth enrolled in HIV care; median age: 20 years (IQR 18–21; range: 15–21)	Exposed n = 584; non-exposed n = 340	Differentiated care for youth: youth-oriented HIV care and treatment services delivered in a youth-specific and youth-friendly clinic.	Family-oriented HIV clinic	LTFU (patient missed last appointment by ≥4 months)	Receiving services from youth-friendly clinic was not associated with LTFU [aHR 1.09, 95% CI (0.80–1.56)].
Mosoko et al., 2011 [56]	Cameroon	Retrospective cohort study	HIV-positive patients initiating ART; median age: 35 years (range: 6 months-73 years)	Exposed n = 1,433; non-exposed n = 1,482	ART price reduction from $27.40 (monthly) to $5.50 for first-line and $51.20 to $12.80 for second-line treatment regimens (reduction was 75–80%) implemented Oct 2004 (Oct 2004-Dec 2005)	Patients enrolled prior to Oct 2004 price reduction (Feb 2002-Sept 2004)	Active in care (patient contact within 91 days)	Probability of remaining active in care was not significantly different between cohorts enrolled before and after price reduction [HR 1.1, 95% CI (0.9, 1.2)] at 15-month follow-up
Djarma et al., 2014 [55]	Chad	Retrospective cohort study	HIV-positive adult patients eligible for ART; median age 32 years (range: 15–76)	Exposed n = 299; non-exposed n = 210	ART provided free of charge and stock outs eliminated so access to ART was continuous (Oct 2009-Nov 2011)	ART not free-of charge and stockouts occurred (Apr 2008-Sept 2009)	LTFU (> 3 months since last scheduled visit)	LTFU was 72.3% before continuous free-of-charge access period and 10% during continuous free-of-charge access period (p<0.001).
Nakiwogga-Muwanga et al., 2015 [57]	Uganda	Prospective cohort study	HIV-positive patients ≥ 18 years who had visited clinic in last 90 days and had appointment scheduled in next 30 days; age range: 25–45+ years	Tracked patients n = 139; patients who resumed care before tracking started n = 117	Patient tracking for patients who missed appointments using phone contact or home visit	Patients who missed appointments and returned to care on their own without tracking	Retention in care (not defined)	39% of traceable patients were retained in care after 18 months follow up, compared with 61% of patients who resumed care before tracking (p = 0.000, as reported by study authors).
Stella-Talisuna et al., 2014 [58]	Uganda	Retrospective cohort study	Adult HIV+ patients receiving support from Reach Out Mbuya; mean age and range not reported	Education support n = 545; food support n = 1637; dual support n = 189	Instrumental support was given to Adult patients with HIV and their families. Patients received one of the following kinds of support: 1. Education support targeting children of HIV patients; 2. Food support targeting food insecure households; 3. Dual support (combination of education and food support) provided to the most vulnerable households based on needs assessment.	N/A	LTFU (no contact with facility for ≥ 90 days after scheduled follow-up date and known not to have died or transferred)	LTFU was 12.3% among education support beneficiaries, 42.1% among food support beneficiaries, and 13.7% dual support beneficiaries.

### Community-based service delivery

Interventions involving the delivery of community-based services had the strongest evidence based on the quality and the number of studies. Eight studies examined shifting the delivery of HIV care and treatment from health facilities to communities [35–42]. These interventions relocated ART distribution to the community and engaged community health workers (CHWs) or peers to distribute ART and monitor symptoms, resulting in fewer patient visits to a clinic. Two community-based service interventions also incorporated directly observed therapy (DOT) or counselling [36, 38]. Four of the interventions were conducted with adults only and four with populations that included adults and adolescents, of which only one reported results for youth separately from adults. Overall, the results of community-based service interventions were positive: six found positive associations between the interventions and retention in care, and two found no association.

The best data for community-based service interventions came from five studies of good or fair quality in Rwanda, Kenya, and South Africa. Two observational studies examined community-based adherence support (CBAS) that consisted of home visits by CHWs who distributed ART and provided psychosocial and health-related support; both showed significant improvements in retention in care. In a retrospective cohort study of adolescents and adults in South Africa, CBAS workers conducted weekly home visits for one month to perform adherence checks and provide psychosocial support; stable patients were then visited every three months [37]. Compared to patients who did not receive CBAS, lower LTFU (no visit to a clinic for ≥180 days) was observed among intervention patients [aHR 0.63 (0.59–0.68)]. A prospective cohort study of adults in their first year on ART in Rwanda found that patients in community-based care had a lower rate of attrition [aHR 0.17 (0.09–0.35)] and were more likely to be retained with a suppressed viral load [aRR 1.15 (1.03–1.27)] one year after initiating ART than were patients in facility-based care [38]. These two studies suggest that CBAS may be useful to improve retention in care, and warrant more rigorous evaluation. A quasi-experimental study of community-based support groups in Kenya—consisting of patient-defined family, friends and other supporters—found that intervention participants had half the rate of clinic absences (≥90-days) in the 22-month period after ART initiation compared to those in the comparison arm [HR 0.48 (0.25–0.92)] [39]. An RCT in Kenya evaluated LTFU among adult patients who received monthly home visits and ART distribution from PLHIV lay workers compared to standard of care (monthly visits to a clinic) [41]. Lay health workers’ home visits were guided by a personal digital assistant (PDA). In this non-inferiority study, the authors found comparable LTFU in the groups over a 12-month period; however, the study was limited by a small sample size and was not powered for this comparison.

Only one study of community-based services evaluated outcomes for youth (16–24 years) separately from adult (≥25 years) participants. This retrospective cohort study in South Africa observed lower LTFU (no visit in >3 months) among patients involved in “community-based adherence clubs” (groups of stable patients led by CHWs, which met bimonthly for counselling and ART distribution) compared to patients who received care through the community health centre [aHR 0.33 (0.27–0.40)] [42]. In sub-group analyses, youth in the clubs had higher overall LTFU than adults and LTFU for youth was not significantly different in the clubs compared to health centres. All told, this intervention may show promise for adults, but provides no evidence to suggest community-based adherence clubs would be useful for youth.

We identified three studies of community-based service interventions that were of poor methodological quality. Of these studies, one found significantly lower non-retention in care for CBAS patients [40] and two found no association with the intervention [35, 36]. In terms of quality, one study lacked sufficient detail on study design and methods to judge the risk of bias for the research [35], one study attempted to draw conclusions about the effect of an individual level intervention based on group-level data (ecological fallacy) [40], and two of the studies lacked adequate measures of exposure and had high rates of missing data [36, 40].

Overall, community-based services show promise for improving retention in care among adults based on several consistently positive observational study results. However, evidence from experimental studies is mixed and the lone RCT of this intervention was underpowered to detect an effect on retention. The only study to investigate differential effects according to the participants’ age found that the intervention was not associated with improved retention in care for youth. More rigorous evaluations of these strategies are warranted, particularly evaluations that include or target adolescents and youth. The community-based service interventions studied frequently included multiple and differing components so it was not possible to identify which components may be associated with retention in care. Finally, several interventions were conducted only among stable patients who were experienced on ART; a remaining question is whether this strategy should apply to patients who are newly initiating ART.

### Decentralization

We identified four retrospective observational cohort studies that examined decentralization of health services; two were good quality and two were fair quality. Two of these studies were conducted with adult PLHIV only [43, 44] and two included adolescents and adults, but did not report results separately for adolescents [45, 46]. The decentralization interventions typically consisted of shifting service delivery for all patients from a higher-tier health facility (such as a regional hospital) to a lower-tier health facility (such as a primary health clinic). The results of these studies were mixed; two studies observed that patients in decentralized services had higher retention compared to patients in centralized services, whereas two other studies found no differences.

One study in Malawi compared attrition rates among PLHIV between a district hospital and ten peripheral health facilities [46]. This study observed significantly lower attrition rates for patients two years after ART initiation in decentralized versus centralized services (9.9 per 100 person-years at decentralized vs. 20.8 per 100 person years at centralized). Similar results were found in a study in Papua New Guinea [43]. The PAPUA model decentralized care from regional hospitals to rural health district facilities and coordinated patient and provider support. When compared to the centralized care, patients engaged in the PAPUA model had a 15% lower rate of attrition during the first four years of ART [HR 0.85 (0.74–0.99)]. One important limitation of this study is that few baseline characteristics of study participants are documented, so the extent to which the two study groups were similar or dissimilar on important characteristics is unknown.

Two other retrospective cohort studies found little or no association between decentralized services and retention in care. A study in Lesotho that compared LTFU among patients followed in hospitals to those followed in primary health centres found no overall differences in LTFU. However, three-year retention was significantly greater among the men who were treated at health centres compared to those who were treated at hospitals [OR 1.53 (1.20–1.96)] in a stratified analysis [45]. A fourth study, in Kenya, compared retention in HIV treatment (the ratio of scheduled monthly visits attended to the number of months in treatment) between patients who chose to access treatment from semi-mobile clinics and patients who accessed treatment from a district hospital [44]. This study also found no differences in retention between study groups; however, those who chose to obtain care in mobile clinics were older, sicker, and poorer than those that chose hospital-based services.

In conclusion, the evidence of an association between decentralized care and improved retention is mixed and limited to retrospective observational studies. None of the studies presented separate results for adolescents. Additional, more rigorous research is needed to examine the effects of decentralized care on retention among PLHIV, and should investigate possible differential effects across sex and age.

### Task shifting and down-referral

Five studies in this review described that shifted health service delivery from higher to lower levels of provider or facility. Task shifting typically involves the delegation of certain components of health services, usually less complex care, to lower cadre health providers, freeing up higher cadre providers to attend to more complex health issues [47]. Down-referral interventions in this review shift the delivery of ART for stable patients from higher-level facilities (such as hospitals) to lower-level facilities (such as primary care clinics), allowing hospital staff to focus on ART initiation and managing complicated cases [48–50]. The down-referral interventions included in the review also often involved task shifting.

For down-referral interventions, the definition of “stable” varied among the studies, but generally included the use of ART for at least 4 months, high CD4 count, low viral load, no opportunistic infections, and demonstrated good adherence to ART. Three good-quality retrospective cohort studies conducted with adults in South Africa were described as down-referral and evaluated the association of down-referral interventions with retention in care [48–50]. Interventions in these studies varied and included components such as shifting care to a group model of service delivery, task-shifting HIV care to lower-level providers, and reducing the frequency of ART pick-up and patient visits to the clinic. Down-referred patients could be referred back to the higher-level facility if they required additional clinical care (e.g. had opportunistic infections). Two studies documented lower LTFU among patients who were down-referred and one study found higher LTFU among down-referred patients compared to those who were not down-referred.

One study compared the retention of ART patients who were down-referred to a group-based model of care (managed by trained counsellors) and patients who received routine nurse-led care in the health clinic [50]. The investigators found that the odds of attrition (LTFU or death) were 57% lower among intervention participants than among clinic-based participants [aHR 0.43 (0.21–0.67)] between 2007 and 2011. A significant association was also observed in a retrospective matched cohort study comparing LTFU between patients who were down-referred to nurse-managed primary health clinics and patients who remained in doctor-managed sites [48]. In this study, LTFU was significantly lower in down-referred patients compared to doctor-managed patients [HR 0.3 (0.2–0.6)] during 12 months of follow-up. However, a third study found that LTFU was higher in patients who were down-referred to nurse-managed services than those in the doctor-managed clinic [aHR 1.36 (1.09–1.69)] [49]. The authors note that the greater LTFU in the down-referral site was associated with male gender, ages 25–34 years, and more advanced HIV disease. LTFU by treatment cohort also increased over time in this study—those who initiated ART in later years were more likely to be lost to follow up than those who initiated ART earlier.

We identified two studies, of good and fair quality, of task shifting interventions that shifted the delivery of HIV treatment and care from physicians or clinical officers to other health care workers, such as nurses [51, 52]. Both studies included adolescents and adults in their study populations, but did not report results separately for adolescents.

The first of these studies produced the most robust evidence with a cluster-randomized controlled trial of an intervention that trained nurses to manage HIV care and treatment in South Africa [51]. Program retention was slightly higher after 12 months for patients newly initiating ART in the nurse-care group than it was in the doctor-care group [RR 1.10 (1.04–1.16)]. However, no difference in program retention was seen for patients who had been on ART for at least six months when the intervention was introduced. The overall retention levels were low in this study—63% in the intervention group and 58% in the control group—which raises questions about the utility of this approach for improving patient retention. The second study, a retrospective cohort study of fair quality, found that patients in Malawi who received joint care from nurses and clinical officers had significantly lower attrition (LTFU and mortality) rates than nurse-managed patients [aIRR 0.54 (0.45–0.65)] [52]. Patients managed by clinical officers only had higher attrition rates than nurse-managed patients [aIRR 3.03 (2.56–3.59)]. However, it is important to note that the type of provider that patients received care from was based on patient clinical characteristics (e.g. clinical officers cared for sicker patients and nurses cared for stable patients).

Evidence on down-referral as a strategy to improve retention in care is limited to a small number of retrospective observational studies with mixed results. It is important to note that down-referral is only implemented for stable patients, so patients initiating ART or with adherence problems are not well-suited for this intervention. Interventions that provide targeted care to adolescents should be tailored to their needs, so formative research must be done to determine whether down-referral is beneficial for experienced, stable adolescent patients. Both studies of task-shifting interventions found moderate effects on retention. Task-shifting HIV care from higher-level to lower-level providers slightly improved retention in one study, but only for patients initiating ART, and the overall retention in care was poor. The co-management of patients in a mixed-care model fared better than either nurse-care or clinical officer-care alone. Because neither study reported results for adolescents separately, the potential effects of these interventions on retention of ALHIV are unclear.

### Differentiated care

Two studies in this review described differentiated care: service-delivery models that are modified to address the specific requirements of a subgroup of clients. A good-quality retrospective cohort study examined an intervention in Kenya that provided differentiated care to patients (≥14 years) with low CD4 counts who were initiating ART [53]. Patients in the “high risk express care” (HREC) group received frequent, brief contacts with nurses during the first three months of treatment (in addition to routine care) in order to identify comorbidities, complications, and reinforce adherence. Those in routine care attended a clinician visit two weeks after initiating treatment and monthly visits thereafter. The study found that HREC patients had lower LTFU compared to similar patients in routine care after a median follow up time of 11 months [aHR 0.62 (0.55–0.70)]. Although the intervention population included adolescents, the results were not stratified by age. The clinics were selected based on their capacity to implement the intervention, potentially limiting the generalizability of findings to other clinic settings. Furthermore, only a subset of the patients who were eligible for HREC was enrolled and the reasons for non-enrolment were not documented. Despite the weaknesses of the study, this approach to differentiated care may hold promise to establish ART adherence and reduce LTFU for patients newly initiating ART. The only study in this review conducted exclusively with youth examined youth-friendly clinic services. This good-quality retrospective cohort study compared LTFU among youths (15–21 years) enrolled in HIV services at a youth-friendly clinic (where 30.4% of the patients were youth) with youths enrolled in care at a family-oriented clinic (where only 3.4% of the patients were youth) [54]. Overall, LTFU among youth was extremely high: 61% of youth were LTFU at the youth-friendly clinic and 51% were LTFU at the family-oriented clinic. The investigators found no association in adjusted analyses between the type of clinic and LTFU. However, these results may have been confounded by differences between the patient populations at the two clinics and the lack of differentiation between patients on ART and pre-ART patients at each clinic. The characteristics of the youth-friendly clinic were not well-described in this article, so it is unclear what elements of youth-friendly services were employed at this clinic.

Differentiated care is a promising strategy to improve retention in care for specific populations, however the evidence is limited. The two interventions described above are unique from each other, and showed mixed effects. Interventions which provide differentiated care to adolescents should be evaluated further. Youth-friendly service interventions are widely recommended and warrant further exploration of their effects on retention in care.

### Free or low-cost ART

Two retrospective cohort studies, both of fair quality, examined trends in retention in care before and after national-level changes in the cost of ART to beneficiaries [55, 56]. Both studies included adults and adolescents, but neither study reported results for adolescents separately. One study, conducted in Cameroon, observed an increase in the number of patients initiated on ART, but no change in retention rates among patients enrolled in HIV care over a 15-month period after a 2004 price reduction of ART as compared to a time period prior to the price reduction [56]. The second study, conducted in Chad, found that free-of-charge and continuous access to ART (October 2009 to November 2011) was associated with more than 60% lower LTFU among patients compared to a period when ART was not free-of-charge and stockouts occurred (April 2008 to September 2009) (p<0.001) [55]. The conflicting results of these two descriptive studies leave important questions. More rigorous research is needed to better understand the impact of cost-reductions on retention in care.

### Patient tracking

We identified one study on patient tracking. This prospective cohort study compared retention rates at 18 months between two groups of adult patients in Uganda who had returned to care after missing appointments by 8 to 90 days [57]. The first group of patients returned to care after being tracked by the clinic through telephone contacts or home visits. The second group of patients had resumed care on their own without being tracked by the program. Eighteen months after returning to care, only 39% of patients who were tracked were retained in care, compared to 61% of the patients who had resumed care on their own. Although this intervention was not effective, the patients in the two groups may have differed simply because one group was motivated to return to care on their own, introducing possible confounding that is not accounted for in the analysis. Further research on patient tracking is needed.

### Instrumental support

One study evaluated the effect of providing instrumental support—tangible forms of social support such as financial assistance, goods, or services—to adult PLHIV. This retrospective cohort study in Uganda evaluated an intervention that provided support based on need to adult PLHIV and their families [58]. The intervention provided either: (1) education support for children in the family; (2) food support for food insecure households; or (3) dual support (education and food) for the most vulnerable households. The authors found that LTFU was lowest (12.3%) among beneficiaries who received only education support, followed by dual support beneficiaries (13.7%); LTFU was highest among beneficiaries who received only food support (42.1%). However, the comparison groups likely differed because the patients were allocated to the support groups based on their needs. The implications of the study’s results are unclear because these differences were not measured. Instrumental support is particularly critical for adolescents given that they may not have skills or resources to meet basic needs. Therefore, the effect of this intervention type warrants further investigation.

### Limitations of the reviewed literature

In general, evidence supporting intervention strategies to improve retention is severely lacking. Although we found a number of good-quality studies in terms of design and implementation, the vast majority used observational designs, most of which were retrospective studies further limited by the availability and potentially the quality of available data. Only three studies employed experimental study designs—one quasi-experimental study and two RCTs. The fair and poor quality studies were plagued by poor descriptions of the designs and methods, dissimilar comparison populations with unadjusted differences, flawed analyses, and inadequate data reporting. Although 12 studies included adolescents, only two studies reported outcomes for youth and no study reported outcomes for adolescents. Community-based service interventions often consisted of multiple components that were not evaluated separately, so it was impossible to discern which elements were most effective.

Moreover, the measurement of retention in care varied across the studies—with wide-ranging definitions of LTFU and the use of assorted follow up times for retention. Most studies looked at retention within the first two years of care, which is problematic because retention continues to decline beyond this period. Finally, none of the studies accounted for patients who cycle in and out of care, a phenomenon that has been documented in LMIC [2].

### Recommendations

Our review identified a few intervention types that show promise for increasing the retention of patients in HIV care. These interventions were conducted among predominantly adult populations, and further investigations are needed with adolescent participants. Interventions that involved the delivery of **community-based services** had the best available evidence base for improving retention in care among adults. Other interventions—**decentralization of health services**, **down-referral of stable patients**, and **task-shifting of services**—also show some promise and warrant further research, especially with adolescents. Observational data also indicate that a **differentiated care** approach may improve retention in care for critical patients and warrants further investigation, though the evidence is limited to a single study and the results were not disaggregated for adolescents. Although this intervention did not target adolescents, the approach of differentiating care and more intensively targeting resources toward patients at high risk of poor outcomes early in their care should be considered for adaptation for adolescents. Finally, despite disappointing results from the one study to evaluate **differentiated care through youth-friendly services**, we believe this intervention merits further investigation due to the fact that the study evaluated here had notable limitations and this intervention is widely recommended and utilized [59, 60]. A strength of all of the recommended interventions is that they operate at the institutional or health service level, which has the potential for greater impact across a population than individual level interventions.

**Free or low cost ART, patient tracking** and **instrumental support** showed mixed associations with retention in care, but these studies were limited in number, design, and quality, so insufficient evidence exists to recommend these interventions. Many interventions consisted of multiple components that were not measured separately; future evaluations should attempt to disaggregate these components to identify the drivers of effective outcomes.

## Conclusion

Despite persistent problems in retaining ALHIV in HIV treatment and care, and international goals to increase the retention of adolescents and youth in care, there is a paucity of interventions targeting this subpopulation. Unfortunately, the interventions in this review were limited in scope and predominantly targeted health service delivery. There are few interventions targeting individual, community or policy level factors to increase retention in care in LMIC, even among adult populations. The strongest evidence, although still limited, supports the delivery of community-based services, but it is unknown if this approach is effective among adolescents. Certain interventions warrant further research with adolescents, such as the decentralization of health services, down-referral of stable patients, task-shifting, and differentiated care. Given that adolescents living with HIV represent an extremely vulnerable population, the results of this review call attention to the huge gap in evidence for interventions that improve their retention in care. The scarcity of interventions being evaluated with adolescent populations and the small number of experimental designs reflects the urgent need for investment and work in this area. Evidence must be generated about whether interventions found to be effective for adults can be scaled up and result in positive outcomes for adolescents and youth. The existing evidence base on retention in care interventions for adolescents is insufficient, and new effective approaches must be identified and tested among adolescents if we are to achieve the 90-90-90 treatment targets in this age group.

## Supporting information

S1 File Search strategy. (DOCX)

S2 File PRISMA checklist. (DOC)
