# Supplementary material for: Improving retention in HIV care among adolescents and adults in low- and middle-income countries: A systematic review of the literature
Source: PLoS One. 2017 Sep 29;12(9):e0184879. doi: 10.1371/journal.pone.0184879 (PMC5621671; doi:10.1371/journal.pone.0184879)
Supplement: S1 File — (DOCX) [file pone.0184879.s001.docx]

# Inclusion and Exclusion Criteria

In order to be eligible for inclusion, a reference must include a **description of an intervention or program aimed to increase ARV adherence or retention in HIV care**. Adherence can be measured in a variety of ways, including pill counts, electronic monitoring (i.e. MEMS caps), pharmacy refills, as well as more distal clinical indicators such as CD4 count and viral load. Similarly, retention in care may include loss to follow up, missed clinic appointments, and other measures. Secondly, the reference must **report quantitative measures of adherence or retention outcomes**. References reporting purely qualitative data were not eligible for inclusion. The exception to this rule is **pilot studies** that evaluated feasibility or acceptability; these references were eligible for inclusion if they did not report the outcomes of interest as long as the pilot was explicitly geared towards increasing adherence or retention in care.

Certain exclusion criteria applied to references in this review. Letters, editorials, conference abstracts, and presentations were excluded on the basis of lacking adequate information on interventions and outcomes. References must be published or available in English. References that did not report primary data, including protocols, literature reviews, and meta-analyses, were not eligible for inclusion. However, these references were reviewed and searched to identify potential sources of primary data that were relevant for inclusion.

Certain references that underwent abstract and full text review were judged to be relevant to the objective of the literature search but not salient to the specific task of designing a pilot intervention. References that contained the following topical areas were set aside for later review: High-income country settings; pre-ART care; Option B-plus; clinical/drug regimen interventions (i.e. switching regimens to increase adherence); PrEP and PEP; HIV testing, ART initiation, and/or linkage to care; and populations who use drugs or are incarcerated.

In order to replicate the dates of the MacPherson literature review, references were eligible for inclusion if they had been published between the dates of 11/20/2010 and 11/20/2015.

# Search Strategy and Review Process: Peer Reviewed Literature

## Part I: Search Strategies

### PubMed

**Date of Search:** 11/20/2015

**Results**: 5004 references

**Search Strategy** (All ages Filters-past 5 years):

We ran the McPherson Pubmed search without adolescent key words for the past five years using the following terms: (hiv[MeSH Terms] OR hiv[tw]) AND (antiretroviral therapy[tw] OR anti-retroviral agents/therapeutic use[Mesh Terms] OR anti HIV agents[MeSH Terms] OR antiretroviral therapy, highly active[MeSH Terms] OR ART[tw] OR ARV*[tw] OR antiretroviral*[tw] OR anti-retroviral[tw] OR HAART[tw] OR cART[tw]) AND ((pre-treatment[tw] OR pretreatment[tw] OR prior to treatment[tw] OR prior-to-treatment[tw] OR pre-ART[tw]) OR (loss to follow-up[tw] OR loss to follow up[tw] OR lost to follow-up[tw] OR lost to follow up[tw] OR loss-to-follow-up[tw] OR lost-to-follow-up[tw] OR loss to retention[tw] OR lost to retention[tw] OR treatment initiation[tw] OR retention OR retain* OR attrition) OR (link*[tw] OR link to care[tw] OR link to treatment[tw] OR linkage to care[tw] OR linkage to treatment[tw] OR link into care[tw] OR linkage into care[tw] OR linkage into care[tw] OR linkage into treatment[tw]) OR (eligibility[tw] OR eligible[tw]OR eligib*)OR (Medication Adherence[MeSH Terms] OR Patient Compliance[MeSH Terms OR adher*[tw] OR complian*[tw] OR comply[tw] OR complied[tw] OR noncomplian*[tw] OR non-complian*[tw] OR non-adher*[tw] OR nonadher*[tw]))

After references were entered into EndNote, documents published outside of the allowed date range were excluded and the references were searched for duplicates.

### Web of Science

**Date of search**: 11/20/2015

**Results**: 4853 references

**Search Strategy** (All ages Filters-past 5 years):

We first ran the McPherson Web of Science search without adolescent key words for the past five years using the following terms: ((“hiv”) OR (“HIV”) OR (“human immunodeficiency virus”) OR (“human immune-deficiency virus”)) AND ((“antiretroviral therapy”) OR (“anti-retroviral”) OR (“ART”) OR (“ARV*”) OR (“HAART”) OR (“cART”)) AND (((“pre-treatment”) OR (“pretreatment”) OR (“prior to treatment”) OR (“prior-to-treatment”) OR (“pre-ART”)) OR ((“loss to follow-up”) OR (“loss to follow up”) OR (“lost to follow-up”) OR (“lost to follow up”) OR (“loss-to-follow-up”) OR (“lost-to-follow-up”) OR (“loss to retention”) OR (“lost to retention”) OR (“treatment initiation”) OR (“retention”) OR (“retain*”) OR (“attrition”)) OR ((“link*”) OR (“link to care”) OR (“link to treatment”) OR (“linkage to care”) OR (“linkage to treatment”) OR (“link into care”) OR (“linkage into care”) OR (“linkage into care”) OR (“linkage into treatment”)) OR ((“eligibility”) OR (“eligible”) OR (“eligib*”)) OR ((“adher*”) OR (“complian*”) OR (“comply”) OR (“complied”) OR (“noncomplian*”) OR (“non-complian*”) OR (“non-adher*”) OR (“nonadher”)))

After references were entered into EndNote, documents published outside of the allowed date range were excluded and the references were searched for duplicates.

## Popline

**Date of Search:** 12/7/2015

**Results:** 3064 references

**Description**: For this search we used terms from the McPherson et al. systematic review, excluding terms specific to adolescents. The search also included controlled search terms (“Keywords” used by Popline.

("HIV" OR "AIDS" OR "Human immunodeficiency virus" OR "HIV infections" OR "persons living with hiv/aids")

AND

("antiretroviral therapy" OR "anti-retroviral agents/therapeutic use" OR "anti HIV agents" OR "antiretroviral therapy, highly active" OR "ART" OR "ARV" or "arvs" OR "antiretroviral" OR "anti-retroviral" OR "HAART" OR "cART"OR "antiretroviral drugs")

AND

("pre-treatment" OR "pretreatment" OR "prior to treatment" OR "prior-to-treatment" OR "pre-ART" OR "loss to follow-up" OR "loss to follow up" OR "lost to follow-up" OR "lost to follow up" OR "loss-to-follow-up" OR "lost-to-follow-up" OR "loss to retention" OR "lost to retention" OR "treatment initiation" OR "retention" OR "retained" OR "retaining" OR "attrition"OR "link" OR "linkage" OR "linked" OR "link to care" OR "link to treatment" OR "linkage to care" OR "linkage to treatment" OR "link into care" OR "linkage into care" OR "linkage into care" OR "linkage into treatment" OR "eligibility" OR "eligible" OR "Medication Adherence" OR "Patient Compliance" OR "adhere" OR "adherent" OR "Adhering" OR "compliance" OR "comply" OR "complied" OR "noncompliant" OR "Non-compliance" OR "non-compliant" OR "noncompliance"OR "non-adherence" OR "non-adherent" OR "non-adhering" OR "nonadherent" OR "Non-adherence" OR "non-adhering" OR "treatment" OR "adherence" OR "user compliance")

**Restrict dates 2010-2015** (Popline only allows you to restrict by year not month, so we manually removed articles/items published before 11/20/2010)

After references were entered into EndNote, documents published outside of the allowed date range were excluded and the references were searched for duplicates.

## Part II: Review Process

References from PubMed, Web of Science and Popline were combined in Endnote for review. A total of 12921 references were imported; 3153 duplicates were immediately identified and 9768 references underwent title review. Of these, 1018 were duplicates and 5020 were deemed ineligible. The remaining 3730 references underwent abstract review.

22 additional duplicates were found during the abstract review. 452 references were judged to be eligible for full text review. However, 137 of these were excluded based on not being relevant to the goals of this systematic review.

# Search Strategy and Review Process: Gray Literature

## Part I: Search Strategies

### USAID DEC

**Date of Search**: 12/11/2015

**Results:** 290 (see below for information on exclusions due to dates outside of the target range)

**Description:** For this search we could use Boolean operators but no controlled search terminology. We conducted this search using a modified version of the McPherson PubMed search strategy, altered only in that words with asterisks were expanded (i.e. adheren* to adherence” OR “adherent” OR “adhering”). Then, we identified potentially relevant articles and scanned the references for DEC Keywords for inclusion. Relevant keywords were included in the search strategy.

**Search strategy:**

("hiv" OR "AIDS" OR "HIV/AIDS") AND ("antiretroviral therapy" OR "antiretroviral therapy (ART)" OR "anti-retroviral agents" OR "anti-HIV agents" OR "antiretroviral therapy" OR "ART" OR "ARV" OR "antiretroviral" OR "anti-retroviral" OR "HAART" OR "cART") AND ("pre-treatment" OR "pretreatment" OR "prior to treatment" OR "prior-to-treatment" OR "pre-ART" OR "loss to follow-up" OR "loss to follow up" OR "lost to follow-up" OR "lost to follow up" OR "loss-to-follow-up" OR "lost-to-follow-up" OR "loss to retention" OR "lost to retention" OR "treatment initiation" OR "retention" OR "retained" OR "retaining" OR "retains" OR "attrition" OR "link" OR "linkage" OR "linked" OR "links" OR "linking" OR "link to care" OR "link to treatment" OR "linkage to care" OR "linkage to treatment" OR "link into care" OR "linkage into care" OR "linkage into care" OR "linkage into treatment" OR "eligibility" OR "eligible" OR "Medication Adherence" OR "Patient Compliance" OR "adherence" OR "adhering" OR "adherent" OR "compliance" OR "complying" OR "compliant" OR "comply" OR "complied" OR "noncompliance" OR "noncomplying" OR "noncompliant" OR "non-compliance" OR "non-complying" OR "Non-compliant" OR "non-adherence" OR "non-adhering" OR "non-adherent" OR "nonadherence" OR "nonadhering" OR "nonadherent")

The search did not restrict for date of publication because some reference documents did not have searchable dates. Therefore, the 594 original documents included documents outside of the valid date range. Of this 594, 304 had dates entered into the database that were prior to November 2010 and were excluded. There were 34 documents with no date listed (either lacking a publication year, or lacking a publication month in 2010 or 2015); these were left in the database. Following title or abstract review, hand searching of potentially relevant documents will be conducted in order to find missing dates. **In total, there were 304 exclusions, leaving 290 documents eligible for title review.**

### AIDSFree

Date of Search: 12/7/2015

Results: 218

**Description**: This search engine does not allow for controlled search terms or Boolean operators. Therefore, we conducted three separate searches each of one key word. Because AIDSFree is focused exclusively on HIV/AIDS, it was not necessary to include HIV-related search terms. All results, regardless of publication date, were included. Documents will be excluded on the basis of date after title review.

Search 1:

Searched “adherence”

Results: 208

Search 2:

Searched “retention”

Results: 112

Search 3:

Searched “linkage”

Results: 177

Then combined results: 497

Date before 2010: 51

Duplicates: 228

Eligible for title review: 218

## Part II: Review Process

References from Popline were combined with the published literature review in Endnote and was reviewed with the published literature.

References from AIDSFree and the USAID DEC were exported into Excel and were reviewed together. 290 references from DEC and 218 references from AIDSFree were combined for a total of **508** references for title review. A double review process was used in which two reviewers independently judged the eligibility of each reference, and discrepancies were resolved before moving on to the next phase of review.
